# High-Fat Diet-Induced Weight Gain, Behavioral Deficits, and Dopamine Changes in Young C57BL/6J Mice

**DOI:** 10.3389/fnut.2020.591161

**Published:** 2021-01-20

**Authors:** Jian Han, Pragya Nepal, Anuoluwapo Odelade, Frederick D. Freely, Destiny M. Belton, Joseph L. Graves, Antoniette M. Maldonado-Devincci

**Affiliations:** ^1^Department of Biology, College of Science and Technology, North Carolina Agricultural and Technical State University, Greensboro, NC, United States; ^2^Department of Psychology, College of Health and Human Sciences, North Carolina Agricultural and Technical State University, Greensboro, NC, United States; ^3^Department of Nanoengineering, Joint School of Nanoscience and Nanoengineering, North Carolina Agricultural and Technical State University, Greensboro, NC, United States

**Keywords:** high-fat diet, motor coordination, sensorimotor, anxiety-like behavior, C57BL/6J mice, dopamine, dopamine D2 receptor, dopamine transporter

## Abstract

Chronic exposure to a high-fat diet (HFD) may predispose individuals to neuropathologies and behavioral deficits. The objective of this study was to determine the temporal effects of a HFD on weight gain, behavioral deficits, and dopamine changes in young mice. One-month old C57BL/6J male and female mice were fed either a control diet (containing 10% calories from fat) or a HFD (containing 45% of calories from fat) for 5 months. Physiological measures such as food consumption, body weight, blood glucose, and behaviors such as motor activity, sensorimotor integration, and anxiety-like behaviors were evaluated monthly. Dopamine (DA), dopamine receptor D2 (DRD2), and dopamine transporter (DT) protein expression levels were measured in the midbrain after 5 months of dietary exposure. Results showed that body weight was significantly greater in the HFD-exposed group compared to the control-group at the end of the 4th month, while food consumption was similar in both groups. For behavioral effects, the HFD group exhibited a significant decrease in motor activity in the open field test after 3 months, and rearing frequency after 4 months of dietary exposure. The HFD group also showed deficits in sensorimotor integration after 3 months. Specifically, chronic HFD exposure increased contact time and time to remove the first adhesive tape in the adhesive-tape removal test (*p* < 0.05). Furthermore, the HFD group showed significant deficits in balance/coordination compared to the control group after 4 months of dietary exposure using the beam traverse test, and increased anxiety-like behavior tested by both the open field and light/dark box tests (*p* < 0.05). Neurochemical measurements showed that HFD-exposed mice had significantly higher midbrain DA and DRD2 protein levels compared to the control group after 5 months of dietary exposure (*p* < 0.05). These results indicate that the impact of HFD on the C57BL/6J mouse strain began at the 3^rd^ month of dietary exposure. Behavioral deficits occurred at a similar time point as increased body weight, at about 3–4 months. Overall, this study provides a critical understanding on how HFD-induced changes in weight gain and behavioral deficits in this strain occur over time. The behavioral changes support the idea that changes also occurred in neurochemical pathways such as dopamine dysregulation.

## Introduction

A high fat diet (HFD) has been shown to increase the risk of neurodegenerative disorders such as Alzheimer's and Parkinson's disease in humans (1, 2). Symptoms of these neurodegenerative diseases include motor and cognitive behavioral deficits resulting from a disruption in the biological functions of neurotransmitters in the brain. Dopamine (DA) is an important neurotransmitter regulating the food eating reward circuit, motor activity, and emotion (3–5). Loss of dopaminergic neurons in the brain, especially in the midbrain including the substantia nigra nuclei, is responsible for reduced motor activity, impaired motor and sensory balance, and abnormal changes to food reward circuitry (6). HFD has been shown to disrupt dopaminergic pathways and generate motor behavioral deficits, but the duration of chronic HFD exposure needed to cause these effects is unclear.

HFD has been shown to impair dopaminergic pathways in various rodent models. For example, HFD-induced insulin resistance slowed down nigrostriatal function and favored loss of DA neurons in mice (7, 8). Mice fed with a HFD show deficits in pre-attentive central information processing (9). Decreased dopamine receptor D2 (DRD2) was observed in HFD and high sugar diet-induced obese rats (10). The C57BL/6J mice fed with a high fat and sugar diet for 16 weeks showed a significant increase of DA release from the striatum, but a much slower uptake of DA compared to controls (11). Tweleve-week-old adult Sprague Dawley rats showed that 2 weeks of HFD consumption disrupted DA networks (9, 12). In comparison to controls, obesity-prone rats exhibited 42% lower striatal DRD2 density and 30% lower total dopamine transporter (DAT) total expression of DAT following 8 weeks on a HFD (13). Collectively, these studies show that chronic intake of a HFD can disrupt dopaminergic function in rodents, but the initial time point when disturbances occur is not clear.

A disruption in dopaminergic function has been positively associated with behavioral deficits (14). For example, injection of DA into the nucleus accumbens restored normal motor activity in rats with reduced motor activity from the injection of 6-hhyroxydopamine causing an 83% DA depletion (15). The loss of dopaminergic neurons and subsequent depletion of DA in the substantia nigra are known to cause the motor deficits observed in Parkinson's disease in both humans and rodent models (16–18). Dopamine D1 and D2 receptors in the hippocampus and amygdala are associated with anxiety-related behaviors. In the case of rodent studies, it has been shown that amphetamine withdrawal results in depression and anxiety-like behavior associated with dysregulation of DA (19, 20). Therefore, behavioral deficits in locomotion and anxiety are potential indicators of altered brain neuro-activity related to DA dysregulation. Other neurochemical pathways related to behavior may also be affected, or it may be HFD-induced obesity itself that is causing the deficits due to increased weight, movement problems, and anxiety.

This study aimed to assess the occurrence of motor behavior deficits over time in a young mice given chronic exposure of HFD. The central hypothesis is that HFD will induce behavioral deficits within 5 months of HFD intake which reflect neurochemical changes in the brain, possibly related to dopaminergic pathways. The choice of a 5-month timeline was based on previous research that showed significant elevations in body weight, glucose, insulin resistance, and adipose tissue weight in C57BL/6J mice after 5 months on a HFD (21). The results from this study will contribute to a greater understanding of when and how HFD contributes to behavioral deficits and changes in brain neurochemistry.

## Materials and Methods

### Animals, Diets and Experimental Design

**Figure 1:**
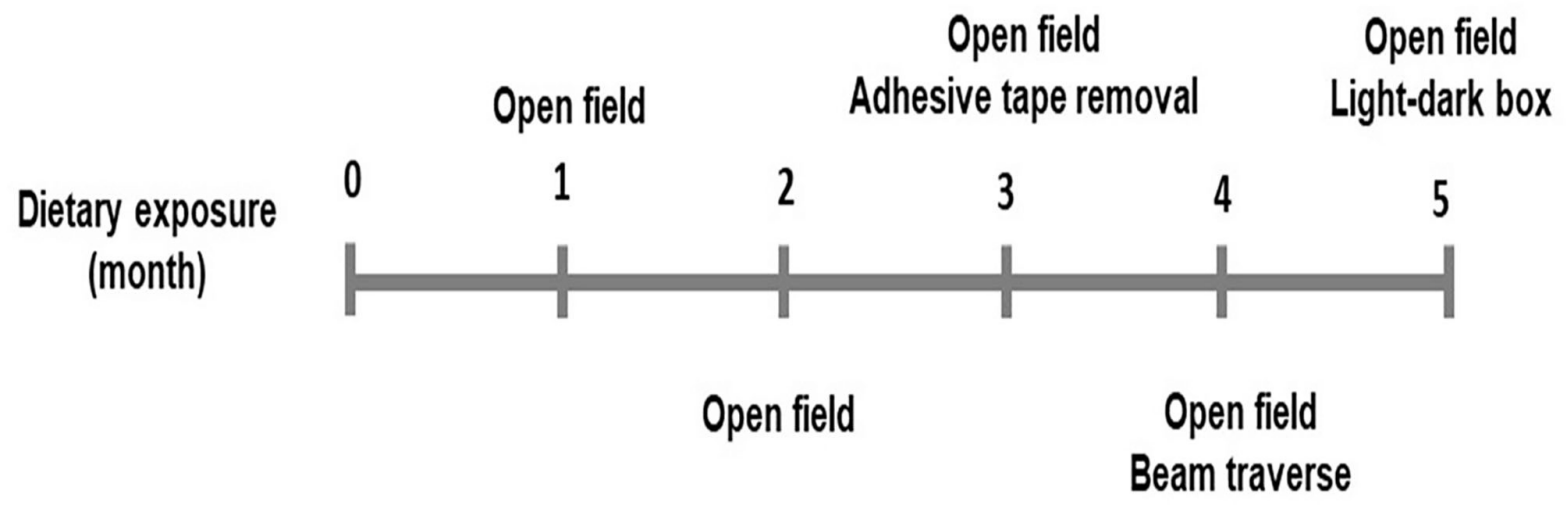
Time points of behavior tests performed.

One-month-old male and female C57BL/6J mice were fed with either a control or a HFD diet (*n* = 15 including 8 male and 7 female for each dietary treatment) for 5 months. Diets were purchased from Research Diets Inc. (New Brunswick, NJ). The energy density for both control (Catalog# D12450H) and HFD (Catalog# D12451) was 4.7 Kcal/g diet. The control diet had 10% calories from the fat, while HFD had 45% calories from the fat. The main source of fat was lard. Mice were housed in individual cages in an animal facility maintained on a 12 h light (7A.M.−7P.M.)/dark cycle and proper temperature control of 24–26°C. Food consumption and body weights were measured weekly. Mice were fasted overnight for 10–12 h and blood glucose was measured from the tail blood using a Relion Prime Blood Glucose Monitoring System (Catalog# 556621084, Walmart, Bentonville, AK). Three categories of behavioral tests for motor activity, sensorimotor integration, and anxiety-like behavior, were measured at the time points shown in Figure 1. All behavioral tests were performed between 9 A.M. and 5 P.M. during the day and estrus was not determined in female mice. Biomedical assays for DA, DRD2, and DAT were performed at the end of the dietary exposure. The animal protocol (#18-006) was proved by the Institutional Animal Care and Use Committee at the North Carolina Agriculture and Technical State University.

### Behavior Analysis

### Motor Activity

#### Open Field Test

The open field test was conducted in Plexiglas chambers (40.6 × 40.6 cm). Motor Monitor software (Kinder Scientific, CA) was used to record motor and anxiety-like activities for 60 min. The total distance traveled and rearing frequencies were recorded as motor activities. Time spent in the center zone, distance traveled, and entries into the center zone were recorded as anxiety-like behaviors (22).

#### Stride Length Test

The stride length test was used to identify gait inconsistency. Reduced length of strides indicate basal ganglia dysfunction (23, 24). The stride length test was conducted separately for the front paw and rear paw. The front and rear paws were painted with red or black ink, respectively. The mice were allowed to walk on a white strip of paper (4.5 cm wide, 40 cm long) placed in an alleyway toward a dark goal box. The distance between the same paw prints were measured, and the four longest distances for each paw were averaged (24).

### Sensorimotor Integration

#### Adhesive Removal Test

The adhesive removal test signifies accurate paw sensitivity and dexterity (25). Two different colored adhesive tapes (5 mm^2^) were placed on the left and right front paws of the mouse. The time taken for the mouse to realize the adhesive tapes were on the paws was recorded as the contact time. The time it took the mouse to remove the adhesive tapes from both paws was recorded as total removal time, which included the contact time. If the mouse did not remove the adhesive tape by 120 s, the trial was terminated and 120 s was recorded as the removal time. There were 20% of HFD mice (*n* = 3) unable to remove the tape, but all control mice removed the tape within 120 s.

#### Beam Traverse Test

The beam traverse test analyzes fine motor coordination and balance in mice (26). The beam contained two sections, with the wider section 12 mm in width and the narrow section 6 mm in width. Each section was 1 m long. The beam was suspended 50 cm above the floor and secured to the tabletop. A black goal box was placed at the narrow end of the beam as the finishing point. The home cage nesting materials were placed in the dark goal box to attract the mouse to walk toward the finish point. A lamp was used to shine light above the starting point and served as an aversive stimulus (26). Mice were placed under the light at the wider end of the beam and trained over three trials to walk across the beam toward the narrow portion of the beam and end at the dark goal box. The fourth trial served as the test trial and was video-recorded for offline analysis. The time taken by the mouse to cross the beam was analyzed for each training trial. For the test trial, the time to cross and number of slips for the wide and narrow portions of the test were analyzed separately.

### Anxiety-Like Behavior

#### Light-Dark Test

The light-dark box tests the levels of exploratory and anxiety-like behaviors expressed by a mouse (27). The light-dark test was conducted in the same apparatus used for the open field test, but with a black plexiglass divider that separated the apparatus into light and dark sections of equal size. The dark box included a door separator and a lamp was used to brightly illuminate the light side. Each mouse was placed in the dark side for 30 s to allow the mouse to acclimate to the dark environment. The separator wall was removed and the mouse was allowed to freely explore both compartments for 10 min. From this test, three parameters were analyzed: time spent by mice in both the light and dark sides, distance traveled by the mice on both sides, and the number of crossovers/entries into the light side. The data were extracted using the Motor Monitor software (Kinder Scientific, CA).

### Brain Dopamine and Related Markers

At the end of the 5th month of dietary exposure, mice were euthanized with CO_2_ followed by decapitation. The brains were removed, and the midbrain region was dissected under a dissection microscope and immediately frozen on dry ice. The midbrain region was processed for ELISA assays to detect DA (Catalog# MBS269234, MyBioSource, San Diego, CA), DRD2 (Catalog# MBS9301506, MyBioSource, San Diego, CA), and DAT (Catalog# MBS2703507, MyBioSource, San Diego, CA) levels following the manufacturer's protocols (28, 29).

### Statistical Analysis

Data were analyzed using two-way ANOVA for Exposure (Control diet, HFD) and Month as a repeated measure, followed by appropriate *post-hoc* tests (Sidak's and Tukey's multiple comparison tests). In cases where behavior and biochemical data were analyzed at one time point, data were analyzed using an independent samples unpaired *t*-test. The level of significance was set at α = 0.05.

## Results

### Food Intake, Body Weight, and Blood Glucose

**Figure 2:**
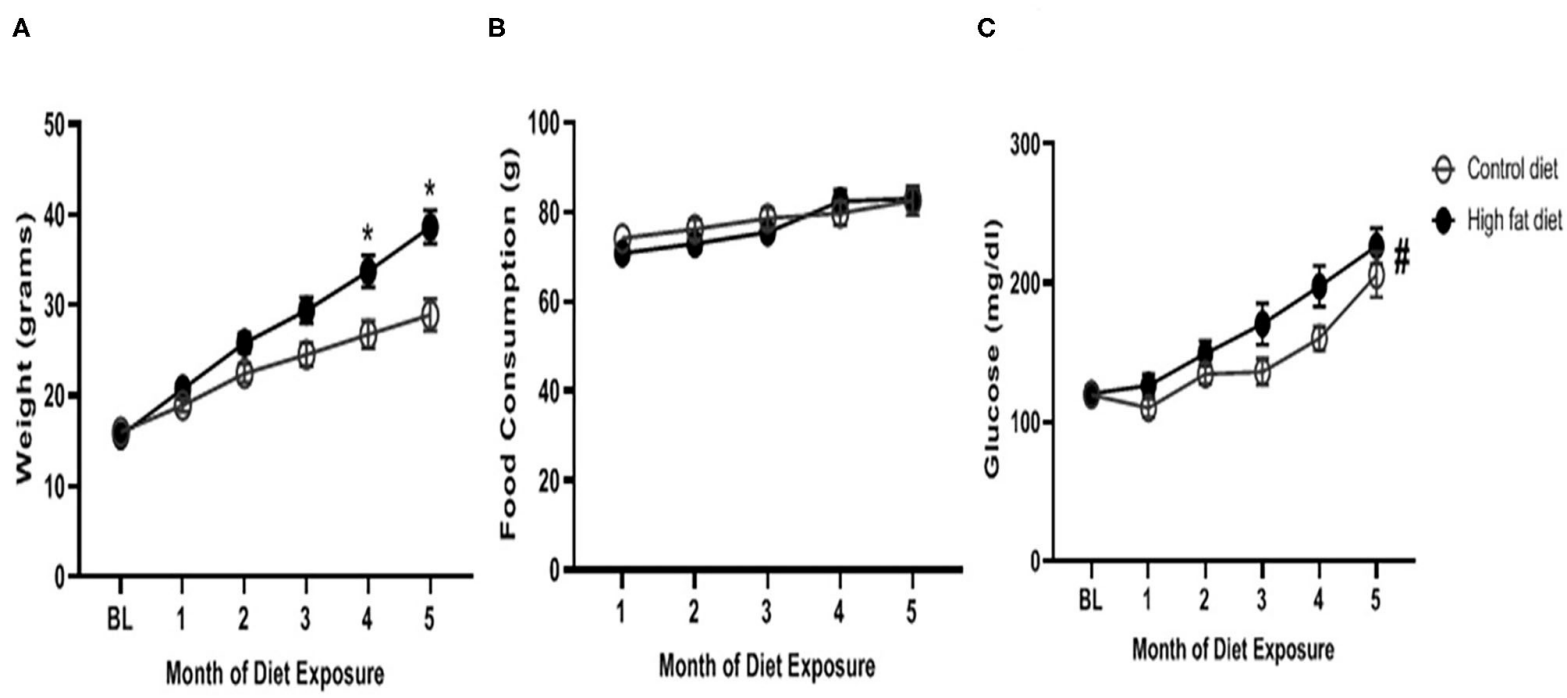
Physiological measures of **(A)** weight, **(B)** food consumption, and **(C)** blood glucose levels across months of exposure to control diet (open circles) and high fat diet (closed circles). Data are presented as mean ± SEM. Control diet-exposed *n* = 15, High fat diet-exposed *n* = 15. * indicates a significant difference between control diet and high fat diet at that month of exposure. # indicates a main effect of diet.

As shown in Figure 2A, HFD-exposed mice began to weigh significantly more than control-diet mice at the 4^th^ and 5^th^ months of dietary exposure as supported by an exposure by month interaction [*F*_(5, 140)_ = 17.21, *p* < 0.0001], and main effects of diet [*F*_(1, 28)_ = 7.93, *p* < 0.001] and month [*F*_(5, 140)_ = 199.20, *p* < 0.0001]. There was a trend for HFD-exposed mice to weigh more at the 3^rd^ month of dietary exposure (*p* = 0.08). As shown in Figure 2B, HFD-exposed mice consumed similar amounts of diet as control-diet exposed mice at each month, however, diet consumption did increase in both groups across months as supported by an exposure by month interaction [*F*_(4, 112)_ = 2.77, *p* < 0.05], and main effect of month [*F*_(2.718, 76.09)_ = 27.97, *p* < 0.0001]. As shown in Figure 2C, blood glucose levels were generally higher in HFD-exposed mice compared to control diet-exposed mice as supported by a main effect of diet [*F*_(5, 140)_ = 31.98, *p* < 0.0001]. Additionally, blood glucose levels increased in both groups over time as supported by a main effect of month [*F*_(1, 28)_ = 5.62, *p* < 0.03]. The diet by month interaction failed to reach statistical significance.

### Behavior Analysis

The HFD group showed significant changes in motor activity, sensorimotor integration, and anxiety-like behavior as described below.

#### Motor Activity

**Figure 3:**
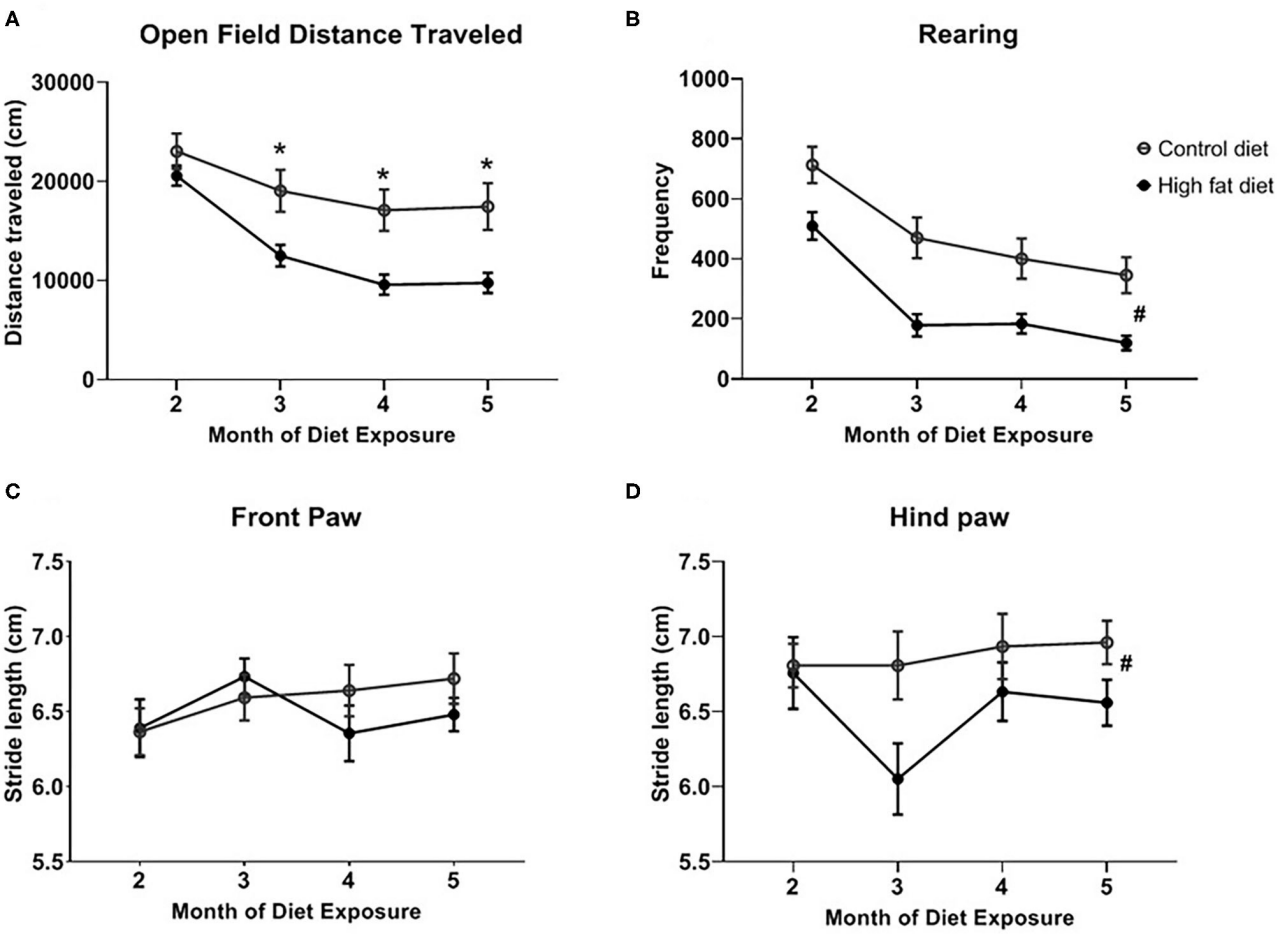
Measures of motor behavior for **(A)** open field distance traveled, **(B)** rearing in the open field test, and stride length for the **(C)** front paw and **(D)** rear paw across months of exposure to control diet (open circles) and high fat diet (closed circles). Data are presented as mean ± SEM. Control diet-exposed *n* = 15, High fat diet-exposed *n* = 15. * indicates a significant difference between control diet and high fat diet at that month of exposure. # indicates a main effect of Diet.

Overall, HFD-exposed mice showed decreased motor behavior across months compared to control-diet exposed mice. As shown in Figure 3A, HFD-exposed mice showed a significant decrease in distance traveled beginning after the 3rd month of exposure as supported by a diet by month interaction [*F*_(3, 84)_ = 10.55, *p* < 0.001], and main effects of diet [*F*_(1, 28)_ = 7.30, *p* < 0.02] and month [*F*_(3, 84)_ = 109.10, *p* < 0.0001]. Rearing behavior decreased more in HFD-exposed mice compared to control-diet exposed mice (Figure 3B) as supported by a main effect of diet [*F*_(1, 28)_ = 13.06, *p* < 0.002] and month [*F*_(3, 84)_ = 73.62, *p* < 0.0001]. The diet by month interaction failed to reach statistical significance. There were no changes in stride length for the front paw across months in either dietary group (Figure 3C) but, in general, HFD exposure decreased stride length for the rear paw (Figure 3D) as supported by a main effect of diet [*F*_(1, 28)_ = 4.89, *p* < 0.05].

#### Sensorimotor Integration

**Figure 4:**
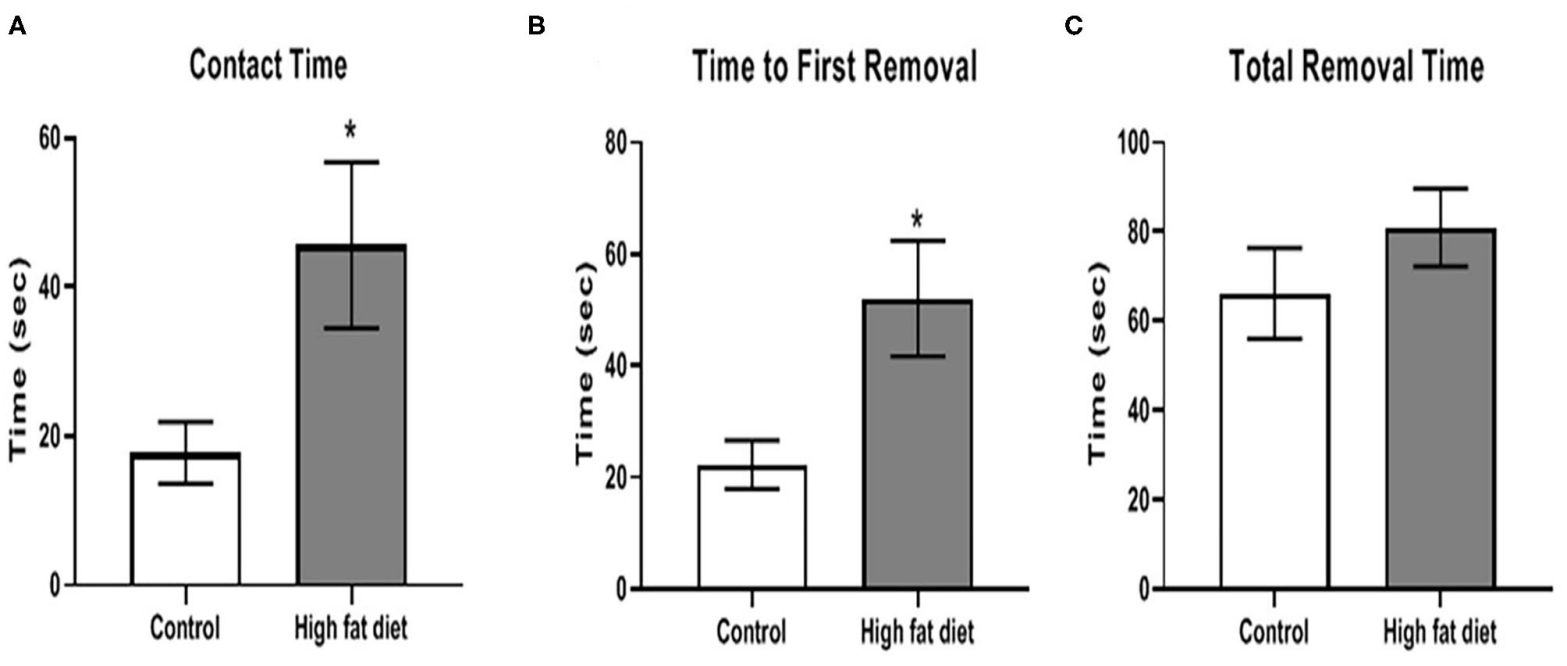
Measures of sensorimotor integration using the adhesive removal test for **(A)** contact time, **(B)** time to removal of first adhesive tape, **(C)** total time to remove both adhesive tapes after 3 months of exposure to control diet (clear bars) and high fat diet (gray bars). Data are presented as mean ± SEM. Control diet-exposed *n* = 15, High fat diet-exposed *n* = 15. * indicates a significant difference between control diet and high fat diet.

A time course for sensorimotor integration was performed but only the significantly different time point was described below. After 3 months of dietary exposure, HFD-exposed mice showed some deficits in sensorimotor integration. Specifically, chronic HFD exposure increased contact time [Figure 4A; *t*(28) = 2.35, *p* < 0.03] and time to remove the first adhesive tape [Figure 4B; *t*(28) = 2.65, *p* < 0.02]. There was no difference in total time, which included the contact time, to remove both adhesive tapes between control and HFD groups (Figure 4C). Twenty percent of the HFD mice reached to maximum testing time of 120 s, and this ceiling effect could contribute to no difference in total time between control and HFD groups. After 3 months, the HFD group tended to show deficits in contact time and total time to remove the first adhesive tape at 4 and 5 months, but the differences between control and HFD groups were not significant (*p* > 0.05).

**Figure 5:**
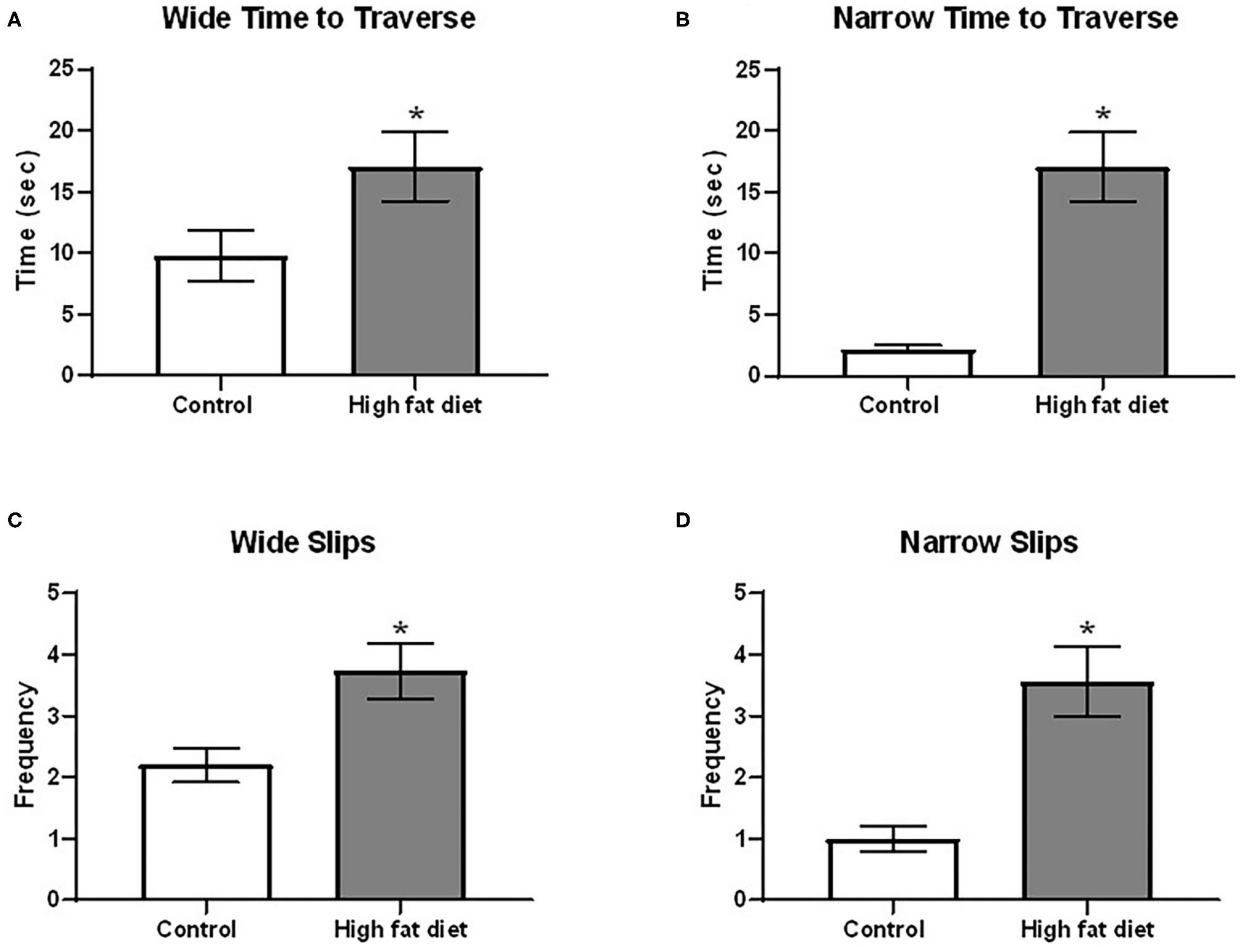
Measures of balance/motor coordination using the beam traverse test following 4 months of exposure to control diet (clear bars) and high fat diet (gray bars). **(A)** time to traverse the wide portion of the beam; **(B)** time to traverse the narrow portion of the beam. **(C)** frequency of slips on the wide portion of the beam; **(D)** frequency of slips on the narrow portion of the beam. Data are presented as mean ± SEM. Control diet-exposed *n* = 15, High-fat diet-exposed *n* = 15. * indicates a significant difference between control diet and high fat diet.

Using the beam traverse test following 4 months of diet exposure, HFD-exposed mice showed deficits in balance/coordination compared to control diet-exposed mice. As shown in Figure 5, HFD-exposed mice showed an increase in time to cross both the wide [Figure 5A; *t*(28) = 2.07, *p* < 0.53] and narrow [Figure 5B; *t*(28) = 5.23, *p* < 0.0001] sides of the beam. Additionally, HFD-exposed mice had a greater frequency of slips on the wide [Figure 5C; *t*(28) = 2.85, *p* < 0.01] and narrow [Figure 5D; *t*(28) = 4.23, *p* < 0.0003] portions of the beam. At the 5^th^ month, the difference between two treatment groups was not significant (*p* > 0.05).

#### Anxiety-Like Behavior

**Figure 6:**
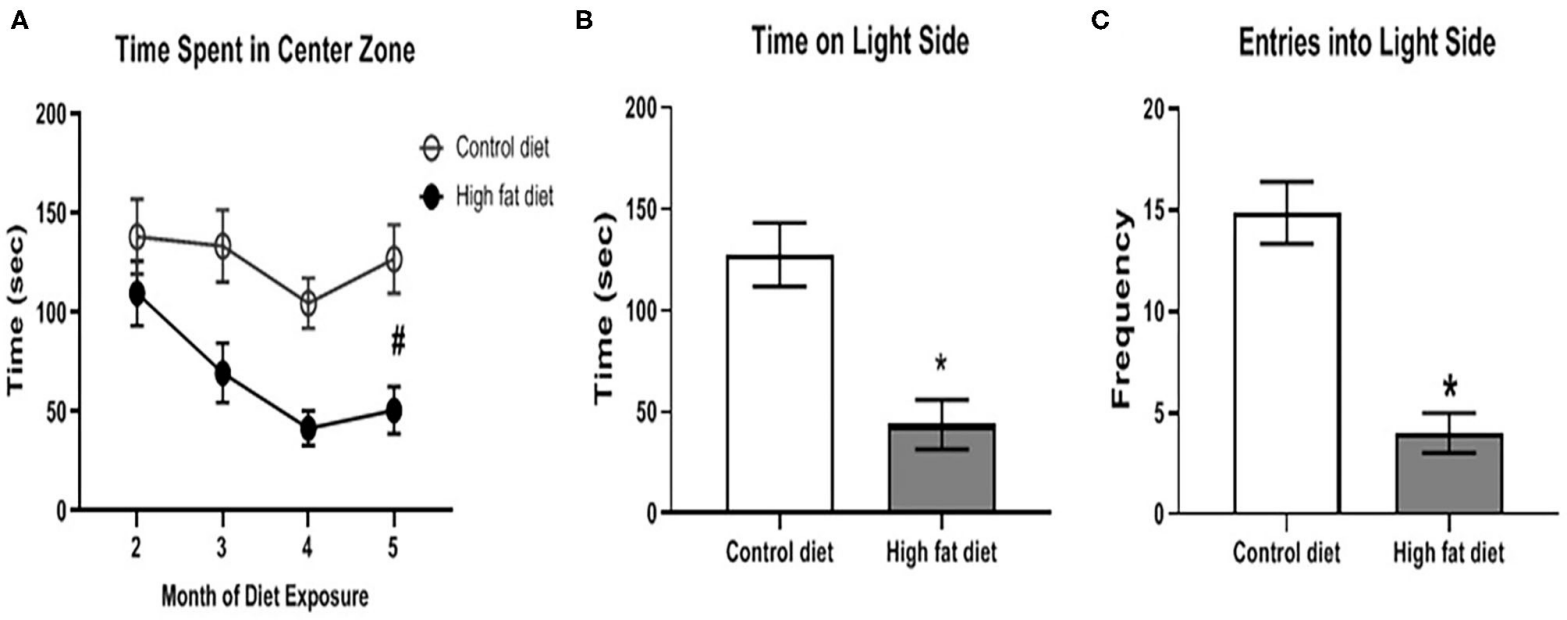
Measures of anxiety-like behavior using the open field test and light/dark test. **(A)** amount of time spent in the center zone of the open field test across months of diet exposure. **(B,C)** amount of time spent on, and number of entries into, respectively, the light side of the two-chambered light/dark test following 5 months of diet exposure. Control diet (open circles/clear bars) and high fat diet (open circles/gray bars). Data are presented as mean ± SEM. Control diet-exposed *n* = 15, High fat diet-exposed *n* = 15. * indicates a significant difference between control diet and high fat diet; # indicates a main effect of diet.

Using the center zone in the open field test and the light/dark test, HFD-exposed mice showed significantly increased anxiety-like behavior compared to control diet-exposed mice after 5 months of dietary exposure. As shown in Figure 6A, HFD-exposed mice showed decreased time in the center zone compared to control diet-exposed mice across months as supported by a main effect of diet [*F*_(1, 28)_ = 13.37, *p* < 0.003] and a main effect of month [*F*_(3, 84)_ = 6.57, *p* < 0.002] at the end of the 5^th^ month of dietary treatment. In the light/dark test, HFD-exposed mice spent less time on the light side [Figure 6B; *t*(26) = 4.11, *p* < 0.0005] and fewer entries into the light side (Figure 6C; *t*(26) = 5.79, *p* < 0.0001) compared to control diet-exposed mice. There was no significant difference in anxiety-like behavior during months 1–4 of the dietary exposure.

### Brain Dopamine and Related Markers

**Figure 7:**
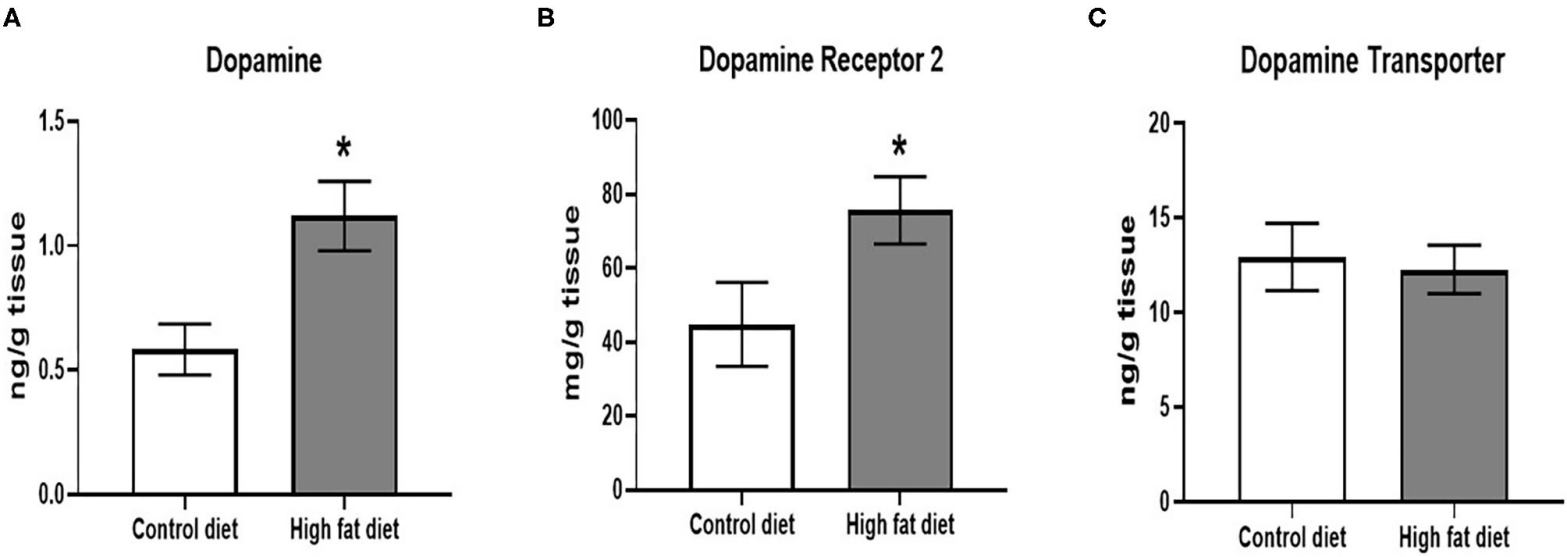
Measures of dopamine and dopamine receptor D2 using ELISA assays. **(A)** amount of dopamine protein per gram of midbrain tissue. **(B)** amount of dopamine receptor D2 protein per gram of midbrain tissue. **(C)** amount of dopamine transporter protein per gram of midbrain tissue. Control diet (clear bars) and high fat diet (gray bars). Data are presented as mean ± SEM. Control diet-exposed *n* = 10, High-fat diet-exposed *n* = 10. * indicates a significant difference between control diet and high fat diet.

Following 5 months of dietary exposure, HFD-exposed mice showed increased DA levels [Figure 7A; *t*(18) = 3.09, *p* < 0.01] and DRD2 receptor levels [Figure 7B; *t*(18) = 2.12, *p* < 0.05]. However, there were no differences in DAT levels between the groups after 5 months of dietary exposure (Figure 7C, *p* > 0.05).

## Discussion

This study aimed to investigate the early changes in behavior and brain dopaminergic function in young mice fed a HFD. Our results showed that initial signs of weight gain and behavioral deficits started at the 3^rd^ month of HFD intake. At the end of the 5th month, the mice fed a HFD showed increased DA and DRD2 levels in the midbrain, but there was no change in DAT levels. This study contributes to the field of nutrition and neuroscience because the identification of early signs of behavioral deficits may reflect initial dysregulation of dopaminergic or other neurochemical pathways, and this information is critical in understanding the progression of brain disorders induced by HFD.

The physiological data collected in this study are consistent with our previous research (21). Previously, we found that 1-month old C57BL/6J mice fed a HFD gained significantly more body weight after 4 months than mice fed the control diet, and also found no difference in food consumption between HFD and control groups and control-diet exposed groups. The increase in body weight on the HFD group is consistent with other reports. A study using young adult C57BL/6J mice (2-month-old) showed significant increases of body weight after 2 months on a HFD (30). Another study found that HFD caused young C57BL/6J mice to weigh 12.4% more than controls after 6 weeks even though the former consumed less food per day (31). Finally, a study using male Wistar rats aged 14–16 weeks also found significant differences in body weight after 4 weeks of HFD exposure (32). These results indicate that rodent models from different age groups chronically fed HFD display greater body weight compared to animals fed control diets. The rate of weight gain on any diet is always a function of age. For example, adult animals might gain weight more quickly due to their slower metabolic rate compared to younger animals (33).

Identifying the initial time point for body weight gain and motor behavioral deficits is fundamental knowledge concerning the impact of HFD in C57BL/6J mice. It may help to reveal underlying conditions of neurophysiology and behavior that are dependent on caloric intake. For example, calorically restricted C57BL/6J mice showed improved cardio-metabolic rates, hippocampus RNA expression, nutrient sensing pathways, age-dependent cognitive function, and dendritic spine density compared to mice fed a control diet (34). In contrast, HFD fed mice showed weight gain, impaired glucose tolerance (IGT), deficits in hippocampal-dependent memory/learning and mood states, and depression-like behavior (31). Results such as these indicate that caloric intake and the time course of weight gain play a crucial role in the etiology of proper brain function (or disorder).

Three categories of behavior were tested in our study as indicators of initial signs of dopamine-related behavioral activities: motor activity, sensorimotor integration, and anxiety-like behavior. Two tests were used in each category to confirm the results, and we found similar times of occurrence for behavioral deficits in each pair of tests. In the open-field test, the HFD-exposed mice showed a decrease in total distance traveled starting at the 3rd month of HFD, and decreased rearing frequency that served as a measure of exploratory behavior. Similarly, in another study, when 8-week-old adult male C57BL/6J mice have been placed on HFD diet for 3 months, they also showed significant decreases in these open-field tasks (30). In our study, both the open-field and the stride length tests support that HFD caused the initial signs of motor deficits beginning at the 3^rd^ month of HFD exposure. These findings are important because deficits in motor activity are one of the important symptoms in many neurodegenerative diseases (35). As we mentioned previously, body weight also increased significantly in HFD mice at the 3rd month of dietary exposure. The motor behavioral deficits and increases in body weight occurred at the same time indicating that these changes may be linked.

Individuals with neurodegenerative disorders often show deficits in sensorimotor integration. Two tests were used to test sensorimotor integration in this study: the adhesive tape removal and beam traverse tests. After 3 months, HFD-exposed mice showed increased time to remove the first adhesive tape from their paws. Other studies have also shown that obesity deteriorates sensorimotor integration. Following the 4th month of dietary exposure in our study, HFD-exposed mice showed deficits in balance/motor coordination in the beam traverse test. The HFD-exposed mice took a longer time to cross the beam and had a higher number of slips than the control group. Mice with destroyed dopaminergic neurons in the substantia nigra by injection of 1-methyl-4-phenyl-1,2,3,6-tetrahydropyridine spent more time crossing the beam compared to control animals (36). These results were also observed in rats. Male Sprague-Dawley rats fed a high fat and sucrose diet performed worse in removing the adhesive tape compared to the control group (37). In male Sprague-Dawley rats fed with a HFD for 3 months, the rats showed significant impairments in the beam-traverse test (38). Overall, these results showed that sensorimotor integration and balance can be disrupted after 3 and 4 months, respectively, of HFD exposure.

Mood states are also impacted by HFD. HFD has been shown to cause an increase in anxiety that is associated with increased body weight and decreased motor activity in mice (39). In adult C57BL/6J mice fed a HFD for 3 months, increased anxiety-like behavior was observed as decreased time spent in the center zone in the open field test (30). Chronic exposure to HFD has also been shown to result in depression-like behavior (31). Similarly, our study showed an increased anxiety-like phenotype demonstrated by decreased time spent in the center zone of the open field test, and less time spent on the light side in the light/dark box test. Although the light-dark box test is commonly used to test anxiety-like behavior, both the open field and light-dark box tests still rely on locomotor exploratory behavior (27). Therefore, an alternative explanation is that the increased anxiety-like behavior, such as less time spent in the light side of the box, was due to decreased locomotor activity induced by chronic exposure to HFD. In the future, other tests of anxiety-like behavior that do not rely on locomotor behavior (e.g., light-enhanced startle response) will be used as better indicators of anxiety.

In summary, our data showed that initial behavioral deficits and increased body weight are observed after 3 months of HFD exposure. Mice given the HFD diet exhibited significant decreases in motor activity and increases in anxiety-like behavior. However, sensorimotor integration and balance did not differ between control and HFD groups until the 4th month of exposure.

We aimed to link the behavioral deficits induced by HFD with a disturbance in midbrain dopaminergic pathways in this study. However, since we only had measurements of dopaminergic molecules at the end of the dietary exposure, we can only suggest that the decreased motor activity, decreased sensorimotor integration, and increased anxiety-like behavior were associated with dopaminergic changes. To verify an association, more time points will be needed for a kinetic study of the expression of dopaminergic molecules in the future. We did find increased DA and DRD2 protein levels in the midbrain at the end of 5^th^ month of HFD exposure, but the expression of DAT did not change compared to controls. A study in adult C57BL/6J mice given a HFD diet for 3 months showed significantly higher levels of DRD2 in the striatum (40). Increased striatal DA levels were also found in adult C57BL/6J mice after 4-months of a high fat and sugar diet (11). These studies supported our findings, but our model is a developmental model starting with younger, normal weight, 1 month old mice. Increased DA expression may have occurred to compensate for the HFD-induced weight gain and potentially rewarding effects of the HFD (6). However, unchanged DAT level along with increased DA and DRD2 levels in our study could imply that the dopaminergic pathway may have begun to show signs of dysfunction.

In conclusion, our study determined that young adult mice fed HFD developed signs of behavioral deficits, especially in motor activity and anxiety-like behavior. This occurs at the 3^rd^ month of HFD exposure in association with increased weight gain. Our findings demonstrate the initial time point of significant body weight and behavioral deficits related to HFD exposure in this strain. Due to the fact that most rodent models are inbred, this initial point might differ by genetic background. However, we predict that our overall finding that early exposure to HFD leads to increased weight and associated deficits in behavior, is robust. In the future, longer exposure to HFD, more extensive biomolecular assays across associated physiological pathways and systems, increased numbers of time points and larger sample sizes, as well as testing different strains of mice will aid in a more robust detection and mechanistic modeling of behavioral deficits. We expect this would further elucidate the potential involvement of DA, DRD2, and DAT in these behavioral deficits.

## Data Availability Statement

The raw data supporting the conclusions of this article will be made available by the authors, without undue reservation.

## Ethics Statement

The animal study was reviewed and approved by Institutional Animal Use and Care Committee (IACUC), North Carolina Agricultural and Technical State University.

## Author Contributions

JH and AM-D designed the experiments, analyzed the data, and wrote and revised the manuscripts. PN who was a graduate student carried out the behavioral experiments, and analyzed the behavioral and physiological data. AO who was a graduate student carried out the biomedical experiments and analyzed the biomedical data. FF and DB were undergraduate students who performed the behavioral experiments and animal husbandry. JG provided general project guidance and support and assistance with the manuscript. All authors contributed to the article and approved the submitted version.

## Conflict of Interest

The authors declare that the research was conducted in the absence of any commercial or financial relationships that could be construed as a potential conflict of interest.

## References

[1] Martin-Jiménez CA Gaitán-Vaca DM Echeverria V González J Barreto GE. Relationship between obesity, alzheimer's disease, and parkinson's disease: an astrocentric view. Mol Neurobiol. (2017) 54:7096–115. 10.1007/s12035-016-0193-8 27796748

[2] Forny-Germano L De Felice FG Vieira MNDN. The role of leptin and adiponectin in obesity-associated cognitive decline and alzheimer's disease. Front Neurosci. (2018) 12:1027. 10.3389/fnins.2018.01027 30692905 PMC6340072

[3] Conde Rojas I Acosta-García J Caballero-Florán RN Jijón-Lorenzo R Recillas-Morales S Avalos-Fuentes JA. Dopamine D4 receptor modulates inhibitory transmission in pallido-pallidal terminals and regulates motor behavior. Eur J Neurosci. (2020) 52:4563–85. 10.1111/ejn.15020 33098606

[4] Grogan JP Sandhu TR Hu MT Manohar SG. Dopamine promotes instrumental motivation, but reduces reward-related vigour. Elife. (2020) 9:e58321. 10.7554/eLife.58321.sa2 33001026 PMC7599069

[5] van Nuland AJ Helmich RC Dirkx MF Zach H Toni I Cools R. Effects of dopamine on reinforcement learning in Parkinson's disease depend on motor phenotype. Brain. (2020) 143:3422–34. 10.1093/brain/awaa335 33147621 PMC7719026

[6] Bissonette GB Roesch MR. Development and function of the midbrain dopamine system: what we know and what we need to. Genes Brain Behav. (2016) 15:62–73. 10.1111/gbb.12257 26548362 PMC5266527

[7] Morris JK Bomhoff GL Stanford JA Geiger PC. Neurodegeneration in an animal model of Parkinson's disease is exacerbated by a high-fat diet. Am J Physiol Regul Integr Comp Physiol. (2010) 299:R1082–90. 10.1152/ajpregu.00449.2010 20702796 PMC2957375

[8] Bousquet M St-Amour I Vandal M Julien P Cicchetti F Calon F. High-fat diet exacerbates MPTP-induced dopaminergic degeneration in mice. Neurobiol Dis. (2012) 45:529–38. 10.1016/j.nbd.2011.09.009 21971528

[9] Labouesse MA Stadlbauer U Langhans W Meyer U. Chronic high fat diet consumption impairs sensorimotor gating in mice. Psychoneuroendocrinology. (2013) 38:2562–74. 10.1016/j.psyneuen.2013.06.003 23850224

[10] Johnson PM Kenny PJ. Dopamine D2 receptors in addiction-like reward dysfunction and compulsive eating in obese rats. Nat Neurosci. (2010) 13:635–41. 10.1038/nn.2519 20348917 PMC2947358

[11] Fritz BM Muñoz B Yin F Bauchle C Atwood BK. A high-fat, high-sugar ‘western’ diet alters dorsal striatal glutamate, opioid, and dopamine transmission in mice. Neuroscience. (2018) 372:1–15. 10.1016/j.neuroscience.2017.12.036 29289718 PMC5809281

[12] Barry RL Byun NE Williams JM Siuta MA Tantawy MN Speed NK. Brief exposure to obesogenic diet disrupts brain dopamine networks. PLoS ONE. (2018) 13:e0191299. 10.1371/journal.pone.0191299 29698491 PMC5919534

[13] Narayanaswami V Thompson AC Cassis LA Bardo MT Dwoskin LP. Diet-induced obesity: dopamine transporter function, impulsivity and motivation. Int J Obes.(2013) 37:1095–103. 10.1038/ijo.2012.178 23164701 PMC3856583

[14] Stojakovic A Walczak M Cieślak PE Trenk A Sköld J Zajdel J. Several behavioral traits relevant for alcoholism are controlled by 2 subunit containing GABAA receptors on dopamine neurons in mice. Neuropsychopharmacology. (2018) 43:1548–56. 10.1038/s41386-018-0022-z 29463910 PMC5957272

[15] Heffner TG Zigmond MJ Stricker EM Effects of dopaminergic agonists and antagonists of feeding in intact and 6-hydroxydopamine-treated rats. J Pharmacol Exp Ther. (1977) 201:386–99. 859104

[16] Golden JP Demaro JA Knoten A Hoshi M Pehek E Johnson EM. Dopamine-dependent compensation maintains motor behavior in mice with developmental ablation of dopaminergic neurons. J Neurosci. (2013) 33:17095–107. 10.1523/JNEUROSCI.0890-13.2013 24155314 PMC3807031

[17] Chen YH Wang V Huang EY Chou YC Kuo TT Olson L. Delayed dopamine dysfunction and motor deficits in female parkinson model mice. Int J Mol Sci. (2019) 20:6251. 10.3390/ijms20246251 31835787 PMC6940785

[18] Paul SS Dibble LE Olivier GN Walter C Duff K Schaefer SY. Dopamine replacement improves motor learning of an upper extremity task in people with Parkinson disease. Behav Brain Res. (2020) 377:112213. 10.1016/j.bbr.2019.112213 31526767 PMC7398159

[19] Cryan JF Hoyer D Markou A. Withdrawal from chronic amphetamine induces depressive-like behavioral effects in rodents. Biol Psychiatr. (2003) 54:49–58. 10.1016/S0006-3223(02)01730-4 12842308

[20] Mogollon R Calil PHR. Counterintuitive effects of global warming-induced wind patterns on primary production in the Northern Humboldt current system. Glob Chang Biol. (2018) 24:3187–98. 10.1111/gcb.14171 29656612

[21] Han J Plummer J Liu L Byrd A Aschner M Erikson KM. The impact of obesity on brain iron levels and alpha-synuclein expression is regionally dependent. Nutr Neurosci. (2019) 22:335–43. 10.1080/1028415X.2017.1387720 29034829

[22] Seibenhener ML Wooten MC. Use of the open field Maze to measure locomotor and anxiety-like behavior in mice. J Vis Exp. (2015) 6:e52434. 10.3791/52434 25742564 PMC4354627

[23] Dutheil S Ota KT Wohleb ES Rasmussen K Duman RS. High-fat diet induced anxiety and anhedonia: impact on brain homeostasis and inflammation. Neuropsychopharmacology. (2016) 41:1874–87. 10.1038/npp.2015.357 26658303 PMC4869056

[24] Pirker W Katzenschlager R. Gait disorders in adults and the elderly: a clinical guide. Wien Klin Wochenschr. (2017) 129:81–95. 10.1007/s00508-016-1096-4 27770207 PMC5318488

[25] Bouet V Boulouard M Toutain J Divoux D Bernaudin M Schumann-Bard P. The adhesive removal test: a sensitive method to assess sensorimotor deficits in mice. Nat Protoc. (2009) 4:1560–4. 10.1038/nprot.2009.125 19798088

[26] Luong TN Carlisle HJ Southwell A Patterson PH. Assessment of motor balance and coordination in mice using the balance beam. J Vis Exp. (2011) 10:2376. 10.3791/2376 21445033 PMC3197288

[27] Takao K Miyakawa T. Light/dark transition test for mice. J Vis Exp. (2006) 13:104. 10.3791/104 18704188 PMC2504462

[28] Hanami K Nakano K Tanaka Y. [Dopamine receptor signaling regulates human osteoclastogenesis]. Nihon Rinsho Meneki Gakkai Kaishi. (2013) 36:35–9. 10.2177/jsci.36.35 23445730

[29] Barker DH Nugent NR Delgado JR Knopik VS Brown LK Lally MA. A genetic marker of risk in HIV-infected individuals with a history of hazardous drinking. AIDS Care. (2017) 29:1186–91. 10.1080/09540121.2017.1291898 28278565 PMC5766029

[30] Sharma S Fulton S. Diet-induced obesity promotes depressive-like behaviour that is associated with neural adaptations in brain reward circuitry. Int J Obes. (2013) 37:382–9. 10.1038/ijo.2012.48 22508336

[31] Wu H Liu Q Kalavagunta PK Huang Q Lv W An X. Normal diet vs high fat diet - a comparative study: behavioral and neuroimmunological changes in adolescent male mice. Metab Brain Dis. (2018) 33:177–90. 10.1007/s11011-017-0140-z 29101600

[32] Maurer L Tang H Haumesser JK Altschüler J Kühn AA Spranger J. High-fat diet-induced obesity and insulin resistance are characterized by differential beta oscillatory signaling of the limbic cortico-basal ganglia loop. Sci Rep. (2017) 7:15555. 10.1038/s41598-017-15872-x 29138510 PMC5686216

[33] Houtkooper RH Argmann C Houten SM Canto C Jeninga EH Andreux PA. The metabolic footprint of aging in mice. Sci Rep. (2011) 1:134. 10.1038/srep00134 22355651 PMC3216615

[34] Wahl D Solon-Biet SM Wang QP Wali JA Pulpitel T Clark X. Comparing the effects of low-protein and high-carbohydrate diets and caloric restriction on brain aging in Mice. Cell Rep. (2018) 25:2234–43.e36. 10.1016/j.celrep.2018.10.070 30463018 PMC6296764

[35] Mann JJ Stanley M Kaplan RD Sweeney J Neophytides A. Central catecholamine metabolism *in vivo* and the cognitive and motor deficits in Parkinson's disease. J Neurol Neurosurg Psychiatry. (1983) 46:905–10. 10.1136/jnnp.46.10.905 6644314 PMC1027603

[36] Liu Q Sang Heon K Yung-Wei S Sok Cheon P Wonwoong L Jongki H. Neuroprotective effects of Suhexiang Wan on the *in vitro* and *in vivo* models of Parkinson's disease. J Tradit Chin Med. (2019) 39:800–8. 32186150

[37] Hoane MR Swan AA Heck SE. The effects of a high-fat sucrose diet on functional outcome following cortical contusion injury in the rat. Behav Brain Res. (2011) 223:119–24. 10.1016/j.bbr.2011.04.028 21549156 PMC3111862

[38] Langdon KD Clarke J Corbett D. Long-term exposure to high fat diet is bad for your brain: exacerbation of focal ischemic brain injury. Neuroscience. (2011) 182:82–7. 10.1016/j.neuroscience.2011.03.028 21435380

[39] Kandola A Vancampfort D Herring M Rebar A Hallgren M Firth J. Moving to beat anxiety: epidemiology and therapeutic issues with physical activity for anxiety. Curr Psychiatry Rep. (2018) 20:63. 10.1007/s11920-018-0923-x 30043270 PMC6061211

[40] Kozuka C Kaname T Shimizu-Okabe C Takayama C Tsutsui M Matsushita M. Impact of brown rice-specific gamma-oryzanol on epigenetic modulation of dopamine D2 receptors in brain striatum in high-fat-diet-induced obesity in mice. Diabetologia. (2017) 60:1502–11. 10.1007/s00125-017-4305-4 28528402 PMC5491592

